# Adsorption of Congo Red and Methylene Blue onto Nanopore-Structured Ashitaba Waste and Walnut Shell-Based Activated Carbons: Statistical Thermodynamic Investigations, Pore Size and Site Energy Distribution Studies

**DOI:** 10.3390/nano12213831

**Published:** 2022-10-29

**Authors:** Lei Zhang, Libin Yang, Jiabin Chen, Wenjun Yin, Yalei Zhang, Xuefei Zhou, Feng Gao, Jiang Zhao

**Affiliations:** 1 State Key Laboratory of Pollution Control and Resources Reuse, College of Environmental Science and Engineering, Tongji University, Shanghai 200092, China; 2 Key Laboratory of Yangtze Water Environment for Ministry of Education, College of Environmental Science and Engineering, Tongji University, Shanghai 200092, China; 3 Shanghai Institute of Pollution Control and Ecological Security, Tongji University, Shanghai 200092, China

**Keywords:** adsorption mechanism, statistical physics modeling, dye, activated carbon, simulation

## Abstract

In this paper, an advanced statistical physics adsorption model (double-layer model with two energies) is successfully established. On the basis of this model, statistical thermodynamic functions (e.g., entropy (*S*), Gibbs free enthalpy (*G*), and internal energy (*E_int_*)), pore size distribution (PSD), and site energy distribution (SED) functions were successfully developed and applied to investigate the adsorption mechanisms of nanopore-structured ashitaba waste-based activated carbons (AWAC) and walnut shell-based activated carbons (WSAC) on Congo red (CR) and methylene blue (MB) dyes in aqueous solutions. Statistical thermodynamic results indicated that the adsorption reactions involved in this study are entropy-increasing, endothermic, and spontaneous in nature. Furthermore, PSD and SED described the heterogeneity of these adsorbents in terms of geometry or structure and energy and illustrated that the aforementioned adsorption processes are endothermic physisorption. All in all, this study contributed to broadening the understanding of the adsorption mechanisms of dye molecules onto biomass-based activated carbons.

## 1. Introduction

Dyes are soluble organic substances that are widely used in various industries such as paper, printing, textiles, leather, food, and cosmetics [1]. At the same time, large amounts of dye wastewater are generated. These dye-bearing effluents pose indirect or direct health risks to plants, animals, and humans, and therefore water pollution caused by dyes is of wide concern [2]. Even though the concentration of dyes in water is very low, they can pose a very serious hazard. Because of the high solubility that dyes exhibit in the aqueous environment, it is difficult to remove them from the water environment by conventional methods [3]. Numerous methods such as adsorption, photolysis, chemical oxidation, and membrane separation have been applied to treat wastewater containing dyes [4,5]. Notably, adsorption is a physicochemical process that features high dye removal efficiency, simple design and operation, low cost, and low impact on the environment [6]. Hydrogels, aerogels, biochars, metal-organic frameworks, activated carbons, and many other novel adsorbents have been prepared for the removal of dyes from aqueous solutions. Among them, biomass-based activated carbons are gaining increasing attention from researchers due to their wide source of raw materials and competitive adsorption properties [7].

The adsorption isotherm not only helps to get the correct information about the equilibrium of the adsorption process but also can be used to describe the adsorption performance of the adsorbent at a constant temperature [8]. A great deal of work has been done by previous researchers on modeling adsorption isotherms, the most representative of which are the isotherm models of Langmuir, Freundlich, Temkin, and Dubinin-Radushkevich [9]. These classical models have been widely used in the understanding of numerous adsorption systems and the elucidation of adsorption mechanisms, but the vast majority of the parameters of these models are only mathematically significant, lacking quantitative interpretation of physicochemical parameters, and most of the work cannot go beyond experimental findings, which are essential to fully understand the adsorption processes and mechanisms [10]. Therefore, the main objective of this study is to propose an appropriate statistical physics adsorption model based on the knowledge of statistical thermodynamics and statistical physics and to understand the adsorption behavior of pollutants on the adsorbent surface with this advanced model.

A published work [7] has performed batch adsorption experiments to quantify the Congo red (CR) and methylene blue (MB) adsorption isotherms (298 K, 308 K, and 318 K), and explained the adsorption mechanism by an advanced statistical physics adsorption model (double-layer model with two energies) and characterization of these adsorbents (nanopore-structured ashitaba waste-based activated carbons (AWAC) and walnut shell-based activated carbons (WSAC)) before and after adsorption. This work has attracted the attention of many researchers after its publication; specifically, as of 23 September 2022, the paper has been cited 204 times based on the Google Scholar platform, 195 times according to the Scopus platform and 159 times according to the Web of Science platform, and this paper has been selected as a highly cited paper or even a hot paper on the Essential Science Indicators platform several times. However, the above-mentioned paper did not involve the analysis of the adsorption mechanism by utilizing the statistical thermodynamic investigations, pore size distribution (PSD), and site energy distribution (SED) theory studies. In order to further improve the content and conclusions of the previous work and to further explore the mechanism of adsorption of CR and MB on AWAC and WSAC from different perspectives, on the basis of the data of the previous paper, the follow-up investigation was continued as it could provide the readers with more new and essential information about the adsorption mechanism from different points of view.

This manuscript was oriented to describe the adsorption mechanism of CR and MB on nanopore-structured AWAC and WSAC by applying statistical physics theories. Specifically, a double-layer model with two energies was first developed through the grand canonical ensemble in statistical physics, and some working hypotheses were assumed. Then, three thermodynamic functions (i.e., entropy (*S*), Gibbs free energy (*G*), and internal energy (*E_int_*)) were derived, calculated, and interpreted via the application of this advanced statistical physics adsorption model to characterize the adsorption process on a macroscopic scale. In addition, PSD and SED were utilized to further investigate the adsorption mechanism from the energy and geometric or structural perspective of the heterogeneity on the surfaces of AWAC and WSAC.

## 2. Materials and Methods

### 2.1. Batch Adsorption Experiments and Fitting Experimental Data

**Figure 1:**
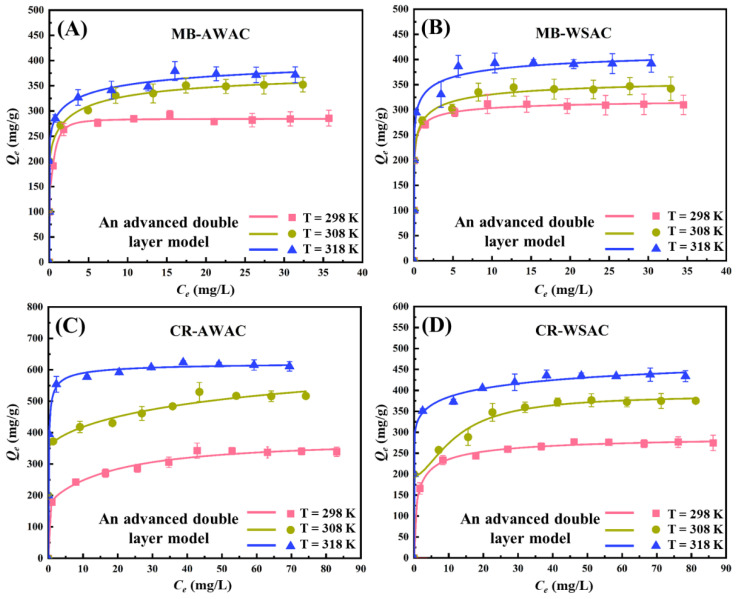
Experimental adsorption isotherms for four adsorption systems (MB-AWAC (**A**), MB-WSAC (**B**), CR-AWAC (**C**) and CR-WSAC (**D**))at 298~318 K, pH = 7, and corresponding fitting curves by using an advanced statistical physics adsorption model (double-layer model with two energies).

Two new promising adsorbents (AWAC and WSAC) were prepared and their effects on the removal of MB and CR dyes were investigated [7]. Experimental CR and MB adsorption isotherms at three operation temperatures (298 K, 308 K, and 318 K) under neutral conditions onto AWAC and WSAC were quantitatively determined, as shown in Figure 1. In addition, these adsorption isotherms were fitted by the Langmuir model, Freundlich model, and an advanced model (double-layer model with two energies), and the results are depicted in Figures S1 and S2, and Figure 1, respectively. With respect to the Langmuir and Freundlich models, the value of the coefficient of determination is low. Considering the advanced statistical physics adsorption model, the coefficient of determination is higher [11], and its corresponding physical parameters are reasonable and simple enough to explain the adsorption mechanism correctly. Overall, the advanced statistical physics adsorption model is the optimal model and was chosen to fit the adsorption data and explain the adsorption mechanism. More details of the experiments have been reported in a previous work [7].

### 2.2. Theoretical Formalism: Double-Layer Model with Two Energies

In this section, we will develop and build an analytical expression for the adsorption isotherm model (double-layer model with two energies) using the statistical physics approach of the giant canonical ensemble and make the following three assumptions:

(a) The adsorption system with determined volume, temperature, and chemical potential allows the system to be studied using the giant canonical ensemble and the results to be interpreted canonically [12,13]. 

(b) The dye molecules adsorbed in an aqueous solution are considered ideal gases, and the interactions between these dissolved molecules that will be adsorbed are so weak that they can be neglected [14,15]. 

(c) The internal degrees of freedom of the adsorbent molecules can be neglected in aqueous solutions, so only the most important degree of freedom, namely the translational degree of freedom, is considered [16,17].

We assume that the *n* dye molecules (*D*) are adsorbed on a receptor site (*R*) and form the *D_n_R* “adsorbent-receptor site” complex, as shown in Equation (1) [18,19]:

(1) $$nD+R⇌D_{n}R$$

If we consider that two layers of adsorbed molecules are formed on the surface of the adsorbent, we specify the first adsorption energy (−*ɛ*_1_) associated with the first adsorption layer, and the second adsorption energy (−*ɛ*_2_) associated with the second adsorption layer. It is worth noting that |*ɛ*_1_| > |*ɛ*_2_| because the energy of direct contact between the dye molecules and the adsorbent is much greater than the interaction energy between the dye molecules [20]. In statistical physics treatment, the microstate of the adsorption process is described by using a giant canonical partition function [18,21]. The receptor site can be empty or occupied by one or more adsorbate molecules. Therefore, we defined *N*_i_ as the occupied state of the receptor site. If the receptor site is not occupied, *N*_i_ = 0. If the receptor site is occupied by *n* or 2*n* molecules, *N*_i_ is 1 or 2, respectively [16]. In this case, the partition function for one receptor site can be written as Equation (2) [22,23].

(2) $$z_{gc}\left(T,\mu \right)=\sum_{N_{i}=0}^{2}e^{-\beta \left(-\varepsilon_{i}-\mu \right)N_{i}}=1+e^{\beta \left(\varepsilon_{1}+\mu \right)}+e^{\beta \left(\varepsilon_{1}+\varepsilon_{2}+2\mu \right)}$$

where (−*ε_i_*) refers to the receptor site adsorption energy (J), *μ* shows the chemical potential of the *D_n_R* complex formed (J), *β* is defined as 1/*k_B_T* where *k_B_* denotes the Boltzmann constant (1.380649 × 10^−23^ J/K), and *T* represents the thermodynamic temperature (K).

Variable numbers of dye molecules are considered to be adsorbed on *D_m_* receptor sites located on a unit mass of adsorbent. The total grand canonical partition function associated with receptor site per surface unit mass (*D_m_*, mg/g), which is hypothesized to be identical and independent, is then written as Equation (3) [23,24].

(3) $$Z_{gc}=\prod_{i=1}^{D_{m}}z_{gc}\left(T,\mu \right)={\left(z_{gc}\left(T,\mu \right)\right)}^{D_{m}}={\left[1+e^{\beta \left(\varepsilon_{1}+\mu \right)}+e^{\beta \left(\varepsilon_{1}+\varepsilon_{2}+2\mu \right)}\right]}^{D_{m}}$$

This total giant canonical partition function allows us to determine the average occupancy number of the receptor sites *N*_0_, which can be written as Equation (4) [25].

(4) $$N_{0}=k_{B}T\frac{\partial lnZ_{gc}}{\partial \mu }=\frac{1}{\beta }\frac{\partial lnZ_{gc}}{\partial \mu }=D_{m}k_{B}T\frac{\partial lnz_{gc}\left(T,\mu \right)}{\partial \mu }$$

The equilibrium adsorption amount (*Q_e_*, mg/g) is expressed as a function of the equilibrium concentration of dye in the aqueous solution (*C_e_*, mg/L) using Equation (5) [26].

(5) $$e^{\beta \mu }=\frac{N}{Z}=\frac{C_{e}}{z}$$

where *N* refers to the number of dye molecules, and *Z* and *z* are the partition function and the partition function per unit volume, respectively.

The chemical potential of the dissolved dye molecules (*μ_m_*, J), using the approximation (b) and (c) can be written as Equation (6) [13,27].

(6) $$\mu_{m}=\frac{\mu }{n}=k_{B}Tln\left(\frac{N}{Z}\right)=k_{B}Tln\left(\frac{N}{Z_{tr}}\right)=k_{B}Tln\left(\frac{C_{e}}{z_{tr}}\right)$$

where *μ* denotes the chemical potential of adsorbed dye molecules, and *Z_tr_* and *z_tr_* are the partition function of translation and the partition function of translation per unit volume, respectively. Here, *z_tr_* can be also expressed as Equation (7) [28,29].

(7) $$z_{\mathrm{tr}}={\left(\frac{2\pi mk_{B}T}{h^{2}}\right)}^{\frac{3}{2}}$$

where *π* = 8.31415, *h* = 6.62607 × 10^−34^ J·s, *m* is the mass of an adsorbed dye molecule (kg).

In addition, the average occupancy number of the receptor sites *N*_0_ is also calculated by Equation (8) [23].

(8) $$N_{0}=D_{m}\frac{e^{\beta \left(\varepsilon_{1}+\mu \right)}+2e^{\beta \left(\varepsilon_{1}+\varepsilon_{2}+2\mu \right)}}{1+e^{\beta \left(\varepsilon_{1}+\mu \right)}+e^{\beta \left(\varepsilon_{1}+\varepsilon_{2}+2\mu \right)}}$$

Additionally, the concentrations at half-saturation for the first and the second layer (*C*_1_ and *C*_2_, mg/L) can be expressed as Equations (9) and (10) [16,23,30].

(9) $$C_{1}=z_{tr}e^{-\beta \varepsilon_{m1}}=z_{tr}e^{\frac{-\Delta E_{1}}{RT}}=C_{s}e^{\frac{-\Delta E_{1}}{RT}}$$

(10) $$C_{2}=z_{tr}e^{-\beta \left(\frac{\varepsilon_{m1}+\varepsilon_{m2}}{2}\right)}=z_{tr}e^{\frac{-\Delta E_{2}}{RT}}=C_{s}e^{\frac{-\Delta E_{2}}{RT}}$$

where *ε_m_*_1_ and *ε_m_*_2_ are the adsorbed molecules’ energies associated with the first and second adsorption layers, respectively; *R* = 8.314 J/(mol·K); *C_s_* denotes the solubility of dye molecules in solution; and Δ*E*_1_ and Δ*E*_2_ are the molar adsorption energies at first and second adsorption layers, respectively.

So, the equilibrium adsorption amount can be expressed as Equation (11) [31].

(11) $$Q_{e}=nN_{0}=nD_{m}\frac{{\left(\frac{C_{e}}{C_{1}}\right)}^{n}+2{\left(\frac{C_{e}}{C_{2}}\right)}^{2n}}{1+{\left(\frac{C_{e}}{C_{1}}\right)}^{n}+{\left(\frac{C_{e}}{C_{2}}\right)}^{2n}}$$

where *n* refers to the number of molecules captured per receptor site.

### 2.3. Statistical Thermodynamic Functions

Based on the knowledge of statistical physics and statistical thermodynamics, thermodynamic properties can be evaluated to enhance the interpretation of the adsorption mechanism. These statistical thermodynamic functions are entropy, Gibbs free enthalpy, and internal energy, respectively.

#### 2.3.1. Entropy

The relationship between the grand thermodynamic potential (*J*), the giant canonical partition function (*Z_gc_*), and the entropy (*S*) is established through Equation (12) [32].

(12) $$J=-k_{B}TlnZ_{gc}=\frac{-\partial lnZ_{gc}}{\partial \beta }-TS$$

The expression for *S* can be obtained by a simple variation of the above equation, as shown in Equation (13) [33].

(13) $$\frac{S}{k_{B}}=-\beta \frac{\partial lnZ_{gc}}{\partial \beta }+lnZ_{gc}$$

With regard to the double-layer model with two energies, the preceding formula can be further specified, and its expression is displayed in Equation (14) [34].

(14) $$\frac{S}{k_{B}}=D_{m}\left\{ln\left[1+{\left(\frac{C_{e}}{C_{1}}\right)}^{n}+{\left(\frac{C_{e}}{C_{2}}\right)}^{2n}\right]-\frac{{\left(\frac{C_{e}}{C_{1}}\right)}^{n}ln{\left(\frac{C_{e}}{C_{1}}\right)}^{n}+{\left(\frac{C_{e}}{C_{2}}\right)}^{2n}ln{\left(\frac{C_{e}}{C_{2}}\right)}^{2n}}{1+{\left(\frac{C_{e}}{C_{1}}\right)}^{n}+{\left(\frac{C_{e}}{C_{2}}\right)}^{2n}}\right\}$$

It is worth noting that *S* reflects the degree of order or disorder of the adsorbent surface during the adsorption of dye molecules. In particular, a positive value of *S* indicates an increase in the degree of disorder of the adsorption system [35].

#### 2.3.2. Gibbs Free Energy

In statistical thermodynamics, the Gibbs free energy (*G*) is usually determined by the chemical potential of the dissolved dye molecules (*μ_m_*) and the equilibrium adsorption amount (*Q_e_*), which can be expressed as Equation (15) [36]:

(15) $$G=\mu_{m}nN_{0}=\mu_{m}Q_{e}$$

where the parameter *μ_m_* can be further expressed as Equation (16) [13].

(16) $$\mu_{m}=k_{B}Tln\left(\frac{C_{e}}{z_{tr}}\right)=k_{B}Tln\left[\frac{C_{e}}{{\left(\frac{2\pi mk_{B}T}{h^{2}}\right)}^{\frac{3}{2}}}\right]$$

For the double-layer model with two energies, the two aforementioned formulas related to the calculation of *G* can be further expressed as Equation (17) [37].

(17) $$\frac{G}{k_{B}T}=ln\left[\frac{C_{e}}{{\left(\frac{2\pi mk_{B}T}{h^{2}}\right)}^{\frac{3}{2}}}\right]\times \left[nD_{m}\frac{{\left(\frac{C_{e}}{C_{1}}\right)}^{n}+2{\left(\frac{C_{e}}{C_{2}}\right)}^{2n}}{1+{\left(\frac{C_{e}}{C_{1}}\right)}^{n}+{\left(\frac{C_{e}}{C_{2}}\right)}^{2n}}\right]$$

Notably, for a specific adsorption system at a given temperature, a negative value of *G* indicates that the removal of adsorbate in this adsorption system is thermodynamically spontaneous [38]. In addition, the more negative the value of *G* demonstrates that the adsorption system is more spontaneous and energetically favorable [39].

#### 2.3.3. Internal Energy

The internal energy (*E_int_*) can also be calculated by the giant canonical partition function (*Z_gc_*), whose formula is presented in Equation (18) [40].

(18) $$E_{\mathrm{int}}=-\frac{\partial lnZ_{\mathrm{gc}}}{\partial \beta }+\mu_{m}N_{0}=-\frac{\partial lnZ_{\mathrm{gc}}}{\partial \beta }+\frac{\mu_{m}}{\beta }\left(\frac{\partial lnZ_{\mathrm{gc}}}{\partial \mu }\right)$$

As for the double-layer model with two energies, the previous formulation for calculating the *E_int_* can be further written as Equation (19) [41].

(19) $$E_{\mathrm{int}}=-D_{m}\left\{\frac{\left(\frac{1}{\beta }\right){\left(\frac{C_{e}}{C_{1}}\right)}^{n}ln{\left(\frac{C_{e}}{C_{1}}\right)}^{n}+2{\left(\frac{C_{e}}{C_{2}}\right)}^{2n}ln{\left(\frac{C_{e}}{C_{2}}\right)}^{2n}}{1+{\left(\frac{C_{e}}{C_{1}}\right)}^{n}+{\left(\frac{C_{e}}{C_{2}}\right)}^{2n}}+k_{B}Tln\left[\frac{C_{e}}{{\left(\frac{2\pi mk_{B}T}{h^{2}}\right)}^{\frac{3}{2}}}\right]\times \left[\frac{{\left(\frac{C_{e}}{C_{1}}\right)}^{n}+2{\left(\frac{C_{e}}{C_{2}}\right)}^{2n}}{1+{\left(\frac{C_{e}}{C_{1}}\right)}^{n}+{\left(\frac{C_{e}}{C_{2}}\right)}^{2n}}\right]\right\}$$

Similar to the case of *G*, if the value of *E_int_* is less than zero, this indicates that the adsorption of dye molecules on the adsorbent surface at a certain temperature is spontaneous [23].

### 2.4. Pore Size Distribution (PSD)

The pore size distribution (PSD) is very important basic information (i.e., the morphology of the AWAC and WSAC adsorbent surfaces) for porous materials such as activated carbons to determine the density of the pores as a function of pore width [42]. While the SED in the following section describes the heterogeneity of the adsorbent surface in terms of energy, here the PSD focuses on the geometric or structural heterogeneity of the adsorbent surface [43]. Statistical physical formalism, Kelvin’s law, and the adsorption isotherm of a double-layer model with two energies were utilized to obtain PSD data, and this method is called the new Kelvin method [44]. Kelvin’s law is given by Equation (20) [45].

(20) $$ln\left(\frac{C_{e}}{C_{s}}\right)=\frac{-2\gamma V_{m}}{rRT}$$

After a simple mathematical transformation, we can also obtain Equation (21) [46]:

(21) $$\frac{C_{e}}{C_{s}}=e^{\frac{-2\gamma V_{m}}{rRT}}=e^{\frac{-K_{k}}{rRT}}$$

where *r* represents the cylindrical pore radius of the adsorbent; *K_k_* = 2*γV_m_* is the Kelvin constant; and *V_m_* refers to the molar volume of the solution, which is calculated from the Equation (22) [47]:

(22) $$V_{m}=\frac{M}{N_{A}\rho }$$

where *M* is the molar mass of adsorbate molecules, *N_A_* shows the Avogadro constant, and *ρ* is the density of the solution.

In addition, *γ* denotes surface tension, which is estimated using the empirical Eötvös equation at three different temperatures, as shown in Equation (23) [48]:

(23) $$\gamma =K_{c}V_{m}{}^{-\frac{2}{3}}\left(T_{c}-T\right)$$

where *K_c_* ≈ 2.1 × 10^−7^ J/(mol^2/3^·K), which is the empirical constant, and *T_c_* is the critical temperature [49].

Then, substituting Equations (24) and (25) into Equation (11), we obtain an equation that relates equilibrium adsorption quantity with the pore radius [44].

(24) $$\frac{C_{e}}{C_{1}}=\frac{C_{s}}{C_{1}}e^{\left(\frac{-K_{k}}{rRT}\right)}$$

(25) $$\frac{C_{e}}{C_{2}}=\frac{C_{s}}{C_{2}}e^{\left(\frac{-K_{k}}{rRT}\right)}$$

Thus, the values of all available parameters except r and the derivative of the equilibrium adsorption amount (*Q_e_*) with respect to the radius (*r*) provide the PSD of these two new adsorbents (AWAC and WSAC), as illustrated in Equation (26) [50,51].

(26) $$\mathrm{PSD}=\frac{dQ_{e}}{dr}=\frac{d\left[n\times D_{m}\times \frac{{\left(\frac{C_{s}}{C_{1}}e^{\left(\frac{-K_{k}}{rRT}\right)}\right)}^{n}+2{\left(\frac{C_{s}}{C_{2}}e^{\left(\frac{-K_{k}}{rRT}\right)}\right)}^{2n}}{1+{\left(\frac{C_{s}}{C_{1}}e^{\left(\frac{-K_{k}}{rRT}\right)}\right)}^{n}+{\left(\frac{C_{s}}{C_{2}}e^{\left(\frac{-K_{k}}{rRT}\right)}\right)}^{2n}}\right]}{dr}$$

The integral of Equation (26) represents the area under the PSD curve and can also characterize the magnitude of saturated adsorption capacity (*Q_sat_*) as shown in Equation (27) [43].

(27) $$Q_{sat}=\int_{0}^{+\infty }{\mathrm{PSD}}_{}\;dr$$

### 2.5. Site Energy Distribution (SED)

One of the most important tools for correlating the evolution of adsorption isotherm parameters values with the evolution of adsorption site energy distribution during adsorption of adsorbate onto the heterogeneous surface of the adsorbent is the “site energy distribution (SED)”, referred to as the “adsorption energy distribution (AED)” [52]. It was found to be useful in providing critical information about the energy distribution of adsorption sites and the degree of heterogeneity of the adsorbent surface [53]. Based on the relationship between the equilibrium adsorption capacity and the energy distribution of the adsorption sites, the general integral isothermal equation of the heterogeneous surface theory of AWAC and WSAC adsorbents can be written as Equation (28) [54].

(28) $$Q_{e}\left(C_{e}\right)=\int_{0}^{+\infty }Q_{h}\left(E,C_{e}\right)\times F{\left(E\right)}_{}dE$$

where *E* refers to the difference between the solute and solvent adsorption energies at a particular adsorption site, *Q_e_*(*C_e_*) is the maximum adsorption amount of adsorbent at the heterogeneous surface of the adsorbate, *Q_h_*(*E, C_e_*) denotes the homogeneous isotherm over local adsorption sites with adsorption energy *E*, and *F*(*E*) represents the frequency distribution of site energies at localized adsorption sites with adsorption energy *E*.

To obtain the site energy distribution, we used the Cerofolini approximation, which gave us the relationship between the equilibrium concentration of the adsorbent (*C_e_*) and the adsorption energy (E^*^), which is shown in Equation (29) [55].

(29) $$C_{e}=C_{s}\times e^{\frac{-E^{*}}{RT}}$$

Incorporating Equations (11) and(29), the double-layer model with two energies isotherm model is expressed as *Q_e_*(E^*^) as shown in Equation(30).

(30) $$Q_{e}\left(E^{*}\right)=n\times D_{m}\times \frac{{\left(\frac{C_{s}\times e^{\frac{-E^{*}}{RT}}}{C_{1}}\right)}^{n}+2{\left(\frac{C_{s}\times e^{\frac{-E^{*}}{RT}}}{C_{2}}\right)}^{2n}}{1+{\left(\frac{C_{s}\times e^{\frac{-E^{*}}{RT}}}{C_{1}}\right)}^{n}+{\left(\frac{C_{s}\times e^{\frac{-E^{*}}{RT}}}{C_{2}}\right)}^{2n}}$$

An approximate SED function *F*(E^*^) can be obtained by bringing the values of all parameters except E^*^ and differentiating the isotherm *Q_e_*(E^*^) with respect to E^*^ [56] (see Equation (31)).

(31) $$\mathrm{SED}=F\left(E^{*}\right)=\frac{-dQ_{e}\left(E^{*}\right)}{dE^{*}}=-\frac{d\left[n\times D_{m}\times \frac{{\left(\frac{C_{s}\times e^{\frac{-E^{*}}{RT}}}{C_{1}}\right)}^{n}+2{\left(\frac{C_{s}\times e^{\frac{-E^{*}}{RT}}}{C_{2}}\right)}^{2n}}{1+{\left(\frac{C_{s}\times e^{\frac{-E^{*}}{RT}}}{C_{1}}\right)}^{n}+{\left(\frac{C_{s}\times e^{\frac{-E^{*}}{RT}}}{C_{2}}\right)}^{2n}}\right]}{dE^{*}}$$

## 3. Results and Discussions

### 3.1. Statistical Thermodynamic Investigations

#### 3.1.1. Entropy

**Figure 2:**
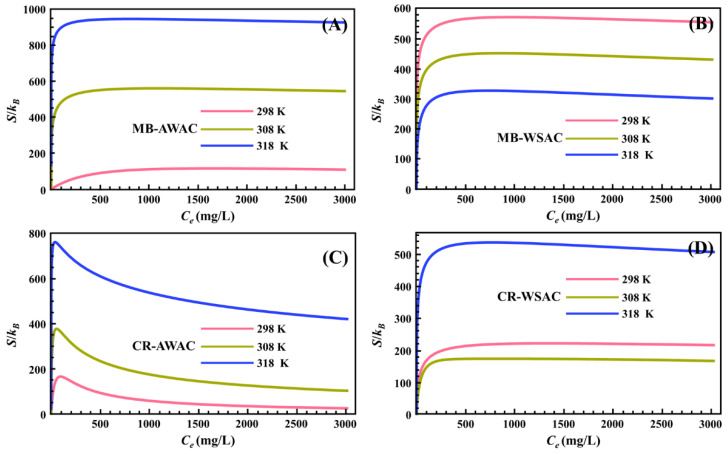
Entropy (*S*) evolution with dye concentration for MB-AWAC (**A**), MB-WSAC (**B**), CR-AWAC (**C**), and CR-WSAC (**D**) adsorption systems.

The variation of parameter *S* with dye concentration at different temperatures is shown in Figure 2. For the four adsorption systems, all *S* values were greater than zero for the range of dye concentrations studied (as depicted in Figure 2), which indicates that the adsorption reactions studied are entropy-increasing reactions and the disorder of the system after the adsorption reaction is greater than the initial state of the adsorption reaction. We also noticed that under the same external conditions, for a particular adsorption system, an increase in temperature significantly boosts its *S* value, mainly due to the fact that the elevated temperature enhances the thermal motion of dye molecules the more disordered the system is [44].

In addition, we found that with a particular concentration (as for this study, this particular concentration is the half-saturation concentration *C*_2_) as the cut-off point, the curves of *S* with a concentration on the left and right sides of that concentration showed two completely different trends [57]. Specifically, before the half-saturation concentration, *S* increases sharply with the increase in dye concentration. At low concentrations, a considerable number of empty active adsorption sites exist on the surface of both AWAC and WSAC adsorbents, so that dye molecules can easily find unoccupied active adsorption sites on the adsorbent surface in a short time, with a consequent rapid increase in the disorder of the system [58]. Particularly, after the half-saturation concentration, *S* decreases slowly with increasing dye concentration. At high concentrations, the number of empty active adsorption sites on the surface of both AWAC and WSAC adsorbents is already quite small, so it is difficult for dye molecules to find unoccupied active adsorption sites on the adsorbent surface and be adsorbed, with a consequent decrease in the disorder of the system [59]. The *S*-value of the entire adsorption system reaches zero when all active adsorption sites on the adsorbent surface are completely saturated [60,61].

#### 3.1.2. Gibbs Free Energy

**Figure 3:**
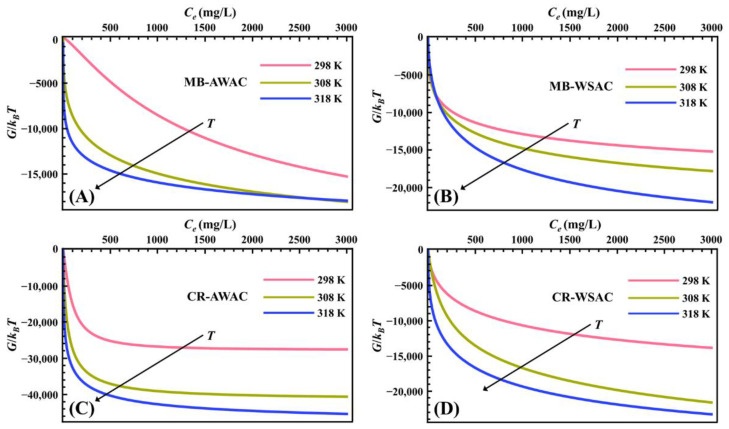
Gibbs free energy (*G*) evolution with dye concentration for MB-AWAC (**A**), MB-WSAC (**B**), CR-AWAC (**C**), and CR-WSAC (**D**) adsorption systems.

Figure 3 illustrates the evolution of parameter *G* with dye concentration at different temperatures. According to Figure 3, all the *G* values are negative, which implies that the MB-AWAC, MB-WSAC, CR-AWAC, and CR-WSAC adsorption systems involved in this study are thermodynamically spontaneous [62,63]. For these four adsorption systems, we found that the absolute value of *G* increases with rising temperature, which on the one hand indicates a higher spontaneity of the adsorption reaction at high temperatures [64], and on the other hand implies a positive correlation between the dye removal capacity and the adsorption temperature [22,65], which further indicates that these adsorption reactions are endothermic [66]. The main reason for the increase in the absolute value of *G* and the endothermic nature is related to the increase in solubility of the MB and CR dye molecules with increasing temperature. This would facilitate the movement of dye molecules in order to reach previously unreachable active adsorption sites on the adsorbent surface [67]. Furthermore, by cross-sectional comparison, we noticed that the absolute value of *G* is the largest for CR-AWAC, followed by CR-WSAC, MB-WSAC, and MB-AWAC at the same temperature and concentration, which is exactly the same pattern as the adsorption amount [68]. This indicates that for this study the adsorption reaction with a large driving force possesses a more excellent adsorption performance [69].

#### 3.1.3. Internal Energy

**Figure 4:**
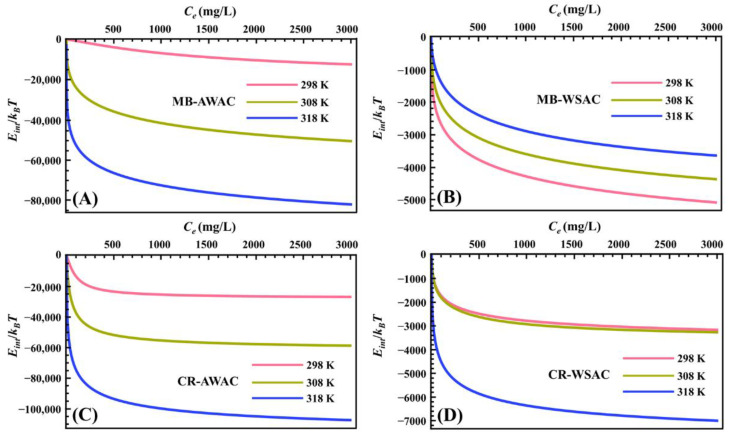
Internal energy (*E_int_*) evolution with dye concentration for MB-AWAC (**A**), MB-WSAC (**B**), CR-AWAC (**C**), and CR-WSAC (**D**) adsorption systems.

The evolution of parameter *E_int_* with dye concentration at different temperatures is exhibited in Figure 4. First of all, we can clearly observe that the *E_int_* value of all adsorption systems is negative, which means that all systems are spontaneous and release energy into the vicinity, reflecting the excellent bonding properties of the MB and CR dye molecules to the AWAC and WSAC surfaces [45,67]. It is also worth mentioning that *E_int_* is significantly lower in the lower range of dye concentrations, indicating that CR and MB dye molecules preferentially adsorb on highly active adsorption sites on the AWAC and CWAC surfaces [70]. When the coverage of CR and MB dye molecules on the adsorbent surface increases (i.e., when the dye concentration increases), CR and MB dye molecules target the less active binding sites, resulting in a slight decrease in the *E_int_* value [71]. Furthermore, the absolute values of *E_int_* show an overall increasing trend with increasing temperature, indicating the interactions between the adsorbed dye molecules on the surfaces of AWAC and WSAC [72].

### 3.2. Pore Size Distribution (PSD) Explorations

**Figure 5:**
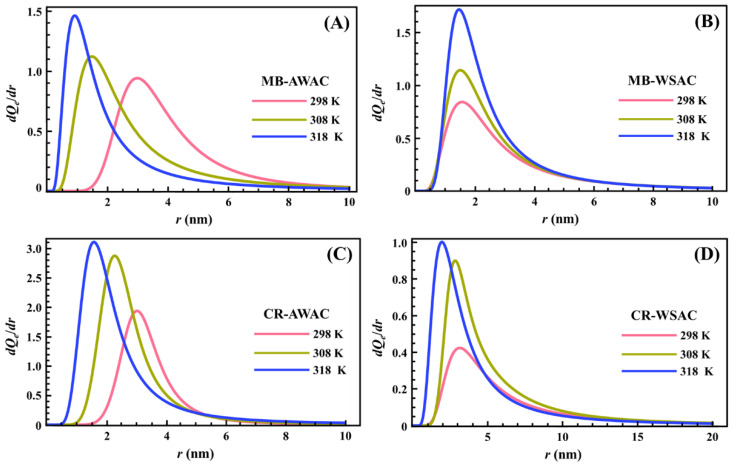
Pore size distribution (PSD) curves of MB-AWAC (**A**), MB-WSAC (**B**), CR-AWAC (**C**), and CR-WSAC (**D**) adsorption systems at different temperatures.

The determined PSDs for the adsorption of two dyes (MB and CR) onto two types of activated carbons (AWAC and WSAC) at different temperatures are depicted in Figure 5. As shown in Figure 5, all PSDs show unimodal distribution, and all the PSDs indicate that the AWAC and WSAC are rich in microporous (*r* < 2 nm) and mesoporous (2 < *r* < 50 nm) structures [73]. The mean values of the cylindrical pore radius obtained from the maximum of the peak are 0.913 nm, 1.463 nm, 1.581 nm, and 1.968 nm for MB-AWAC, MB-WSAC, CR-AWAC, and CR-WSAC, respectively. Moreover, the area between the PSD curve and the *x*-axis represents the magnitude of the saturated adsorption capacity, and we can see that for the four adsorption systems, the saturated adsorption capacity increases with increasing temperature, which further reflects that the four adsorption processes studied in this paper are endothermic adsorption reactions [74]. Furthermore, the increase in temperature leads to a shift in radius toward the low values and an increase in the width of these peaks. These two characteristics are specific to a thermal agitation effect [15,75]. This demonstrates that the increase in temperature promotes an increase in the free motion speed of the dye molecules so that the dye molecules can easily reach smaller pores and can be detected at a smaller pore radius. This variation in distribution may be due to changes in the interaction of the adsorbed probe molecules with the pore surface rather than actual changes in the pore such as pore expansion or contraction, although the thermal expansion of the pores contributes to the easy activation of smaller pores [46].

### 3.3. Site Energy Distribution (SED) Studies

**Figure 6:**
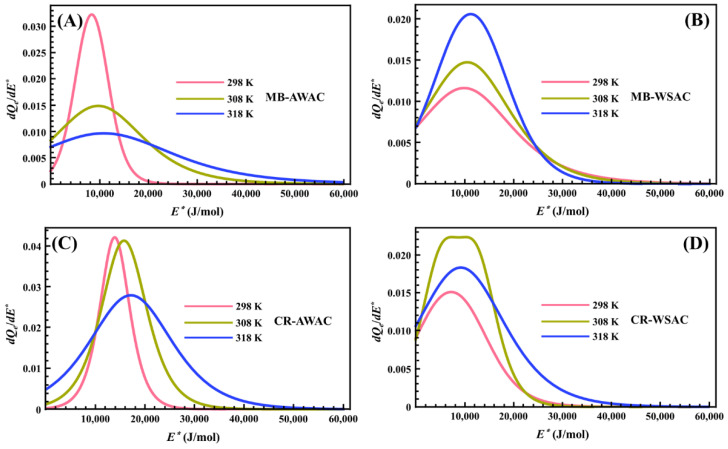
Site energy distribution (SED) curves of MB-AWAC (**A**), MB-WSAC (**B**), CR-AWAC (**C**), and CR-WSAC (**D**) adsorption systems at different temperatures.

Figure 6 represents the behavior of the site energy distribution (SED) involved in the MB and CR adsorption processes onto AWAC and WSAC at three different temperatures. From this figure, we can note that the heterogeneity of the AWAC and WSAC surfaces with respect to the energy of the MB and CR dye molecules on the adsorption sites is vividly described by eleven pseudo-Gaussian peaks and one bimodal peak. After precise measurements, we found that the E^*^ values corresponding to the peak values of these eleven pseudo-Gaussian peaks exactly match the Δ*E_2_* values obtained by previous calculations. Meanwhile, the *E*_1_^*^ and *E*_2_^*^ values corresponding to the two peak values of a bimodal peak coincide with the Δ*E*_1_ and Δ*E*_2_ values. Furthermore, from Figure 6, we can observe that the adsorption energy range of MB and CR dye molecules on each activated carbon does not exceed 60 kJ/mol, for high energies, thus indicating that the adsorption processes involved in this study are related to physical forces rather than chemical forces [76,77]. More specifically, the Van der Waals interactions (4.19~8.37 kJ/mol), hydrophobic bonding forces (around 4 kJ/mol), hydrogen bonding connections (about 2~40 kJ/mol), dipole bond forces (approximately 2~29 kJ/mol), π-π stacking interactions (lower than 10 kJ/mol), and electrostatic interactions (10~50 kJ/mol) can be predicted by interacting the dye molecules with the AWAC and WSAC [78,79,80]. Regarding the effect of temperature on the SED curves, we note that an increase in temperature causes the peaks of the SED curves to shift towards higher energy values. This is consistent with the physical effect of temperature since an increase in temperature implies an increase in the average kinetic energy of the dissolved dye molecules. Further, the increase in temperature also leads to a broadening of these peaks’ widths. This is due to the activation of lower and higher energies by thermal stirring to broaden these peaks’ widths [46,75].

## 4. Conclusions

An analytical expression for the adsorption isotherm model (double-layer model with two energies) was established by using the statistical physics method of the giant canonical ensemble. Subsequently, based on this model and statistical physics knowledge, expressions for statistical thermodynamics (e.g., *S*, *G*, and *E_int_*), PSD, and SED functions were also developed and successfully applied to reveal the adsorption mechanisms of nanopore-structured AWAC and WSAC for MB and CR dyes in aqueous solutions. Studies on *S* revealed that the studied adsorption reactions are entropy-increasing reactions, with *S* reaching a maximum when the dye concentration is at half-saturation concentration (*C*_2_) and reaching zero when the adsorption is saturated. Moreover, the *G* and *E_int_* values suggested the endothermic and spontaneous nature of the adsorption process of MB and CR dye molecules on AWAC and WSAC. Furthermore, PSD and SED described the heterogeneity of these adsorbents in terms of geometry or structure, and energy. Specifically, the PSD explorations confirmed that the four adsorption systems studied in this paper are endothermic adsorption reactions. SED studies demonstrated that the adsorption of MB and CR dye molecules on AWAC and WSAC was accomplished by physical forces, in which Van der Waals, hydrophobic bonding, hydrogen bonding, dipole bonding forces, π-π stacking, and electrostatic interactions can be predicted. Additionally, due to the thermal effect of temperature, an increase in temperature leads to a shift in radius and energy toward the lower and higher values, respectively, and increases the width of these peaks. Overall, these theoretical results provide newer insights into the dye adsorption mechanisms based on the original foundations.

## Data Availability

Not applicable.

## Supplementary Materials

The following supporting information can be downloaded at: https://www.mdpi.com/article/10.3390/nano12213831/s1, Figure S1: Experimental adsorption isotherms for four adsorption systems (MB-AWAC (A), MB-WSAC (B), CR-AWAC (C) and CR-WSAC (D)) at 298~318 K, pH = 7, and corresponding fitting curves by Langmuir model; Figure S2: Experimental adsorption isotherms for four adsorption systems (MB-AWAC (A), MB-WSAC (B), CR-AWAC (C) and CR-WSAC (D)) at 298~318 K, pH = 7, and corresponding fitting curves by Freundlich model.
