# Supplementary material for: Adsorption of Congo Red and Methylene Blue onto Nanopore-Structured Ashitaba Waste and Walnut Shell-Based Activated Carbons: Statistical Thermodynamic Investigations, Pore Size and Site Energy Distribution Studies
Source: Nanomaterials (Basel). 2022 Oct 29;12(21):3831. doi: 10.3390/nano12213831 (PMC9657552; doi:10.3390/nano12213831)
Supplement: Supplementary file 1 [file nanomaterials-12-03831-s001.zip › nanomaterials-1959383-supplementary.pdf]

nanomaterials

Supplementary Materials

Adsorption of Congo Red and Methylene Blue onto Nanopore-structured Ashitaba Waste and Walnut Shell-Based Activated Carbons: Statistical Thermodynamic Investigations, Pore Size and Site Energy Distribution Studies

Lei Zhang [1,2], Libin Yang [1,2,3], Jiabin Chen [1,2,3], Wenjun Yin [1,2], Yalei Zhang [1,2,3], Xuefei Zhou [1,2,3], Feng Gao [1,2,3,*] and Jiang Zhao [1,2,3,*]

1 State Key Laboratory of Pollution Control and Resources Reuse, College of Environmental Science and Engineering, Tongji University, Shanghai 200092, China
2 Key Laboratory of Yangtze Water Environment for Ministry of Education, College of Environmental Science and Engineering, Tongji University, Shanghai 200092, China
3 Shanghai Institute of Pollution Control and Ecological Security, Tongji University, Shanghai 200092, China
* Correspondence: gaofeng_1111@126.com (F.G.); jiangzhao@tongji.edu.cn (J.Z.)

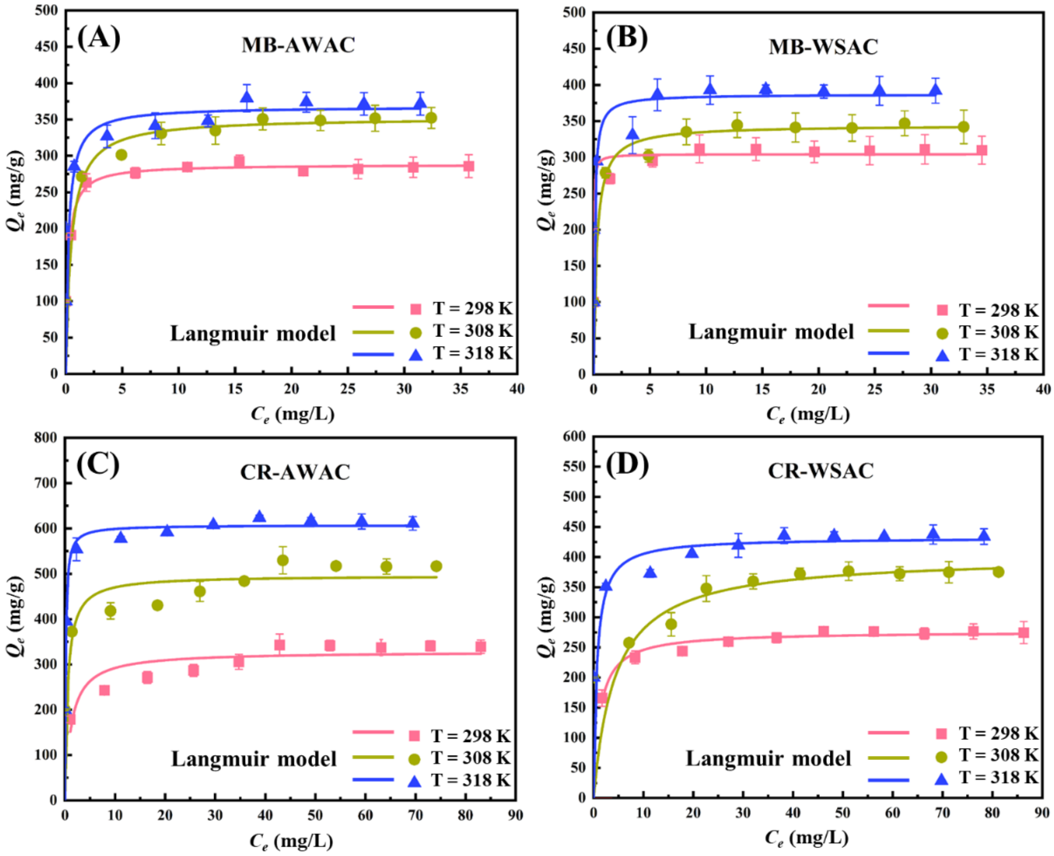

Figure S1. Experimental adsorption isotherms for four adsorption systems (MB-AWAC (A), MB-WSAC (B), CR-AWAC (C) and CR-WSAC (D)) at 298~318 K, pH=7, and corresponding fitting curves by Langmuir model.

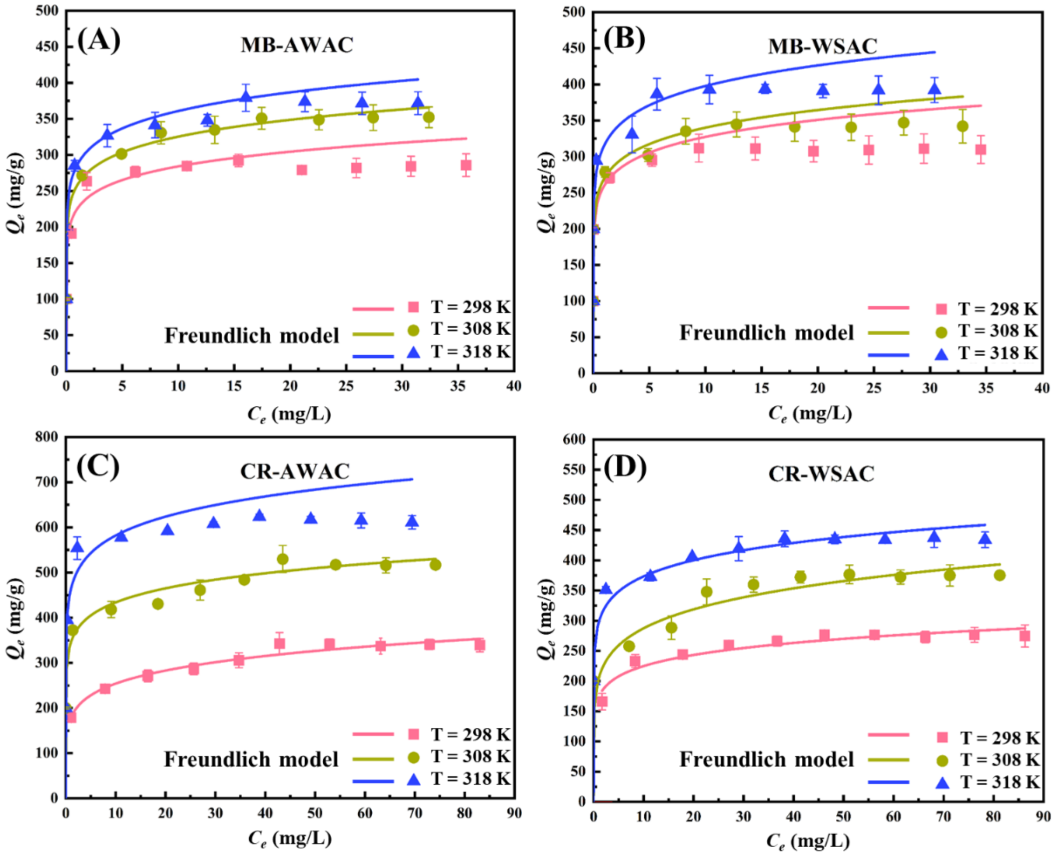

Figure S2. Experimental adsorption isotherms for four adsorption systems (MB-AWAC (A), MB-WSAC (B), CR-AWAC (C) and CR-WSAC (D)) at 298~318 K, pH=7, and corresponding fitting curves by Freundlich model.
